# Stage-Specific Coordination of Auxin, Abscisic Acid, and Sucrose Signaling Controls Cell Expansion and Anthocyanin Biosynthesis in Strawberry

**DOI:** 10.3390/plants15213314

**Published:** 2026-10-26

**Authors:** Ling Ma, Zhenwu Zhang, Chenghua Xing

**Affiliations:** College of Agriculture, Jinhua University of Vocational Technology, Jinhua 321017, China; maling@jhc.edu.cn (L.M.); 20221069@jhc.edu.cn (Z.Z.)

**Keywords:** strawberry, abscisic acid, auxin, sucrose, growth and development

## Abstract

Strawberry (*Fragaria* × *ananassa*) is a typical non-climacteric fruit whose development and ripening are coordinately regulated by auxin (IAA), abscisic acid (ABA) and sucrose signaling. However, stage-specific interactions among these signals in controlling cell expansion and anthocyanin biosynthesis remain incompletely understood. In this study, ‘Sweet Charlie’ strawberry fruits at six developmental stages (small green, SG; medium green, MG; large green, LG; white, WT; initial red, IR; and full red, FR) were treated with exogenous IAA, ABA or sucrose while remaining attached to the plant. Cellular morphology, endogenous hormone profiles, fruit coloration and anthocyanin content, firmness and cell-wall enzyme activities, soluble sugar and organic acid composition, and the expression of key genes involved in anthocyanin biosynthesis, sugar metabolism and ripening were systematically analyzed. Results showed that IAA maintained compact cell arrangement, delayed tissue loosening and softening, suppressed anthocyanin accumulation and the expression of anthocyanin biosynthetic genes (*FaCHS*, *FaCHI*, *FaF3H*, *FaDFR*, *FaANS*, *FaUFGT*, etc.), and limited sugar accumulation across stages. In contrast, ABA and sucrose treatments promoted cell expansion and intercellular space enlargement (especially from WT to FR), accelerated softening by elevating polygalacturonase (PG), pectin methylesterase (PME) and cellulase activities, markedly increased anthocyanin content and CIRG values, enhanced soluble sugar accumulation, and upregulated anthocyanin pathway, sugar metabolism/transport (*FaSPS1*, *FaSUTs*) and ripening-related genes (*FaNCEDs*, *FaSnRK2.6*, etc.). Endogenous hormone dynamics further confirmed opposing patterns between IAA and ABA, with sucrose producing parallel effects with ABA. Collectively, these findings demonstrate that early high IAA levels drive cell expansion while repressing ABA and ripening programs, whereas the subsequent decline of IAA and rise of ABA and sucrose coordinately trigger anthocyanin biosynthesis, sugar accumulation and softening. This work elucidates the stage-specific coordination of IAA–ABA–sucrose signaling in strawberry fruit development and provides a theoretical basis for precise quality regulation of non-climacteric fruits.

## 1. Introduction

Strawberry (*Fragaria* × *ananassa* Duch.) is an economically vital fruit crop worldwide and serves as a classic model for non-climacteric fleshy fruits. Rich in vitamin C, minerals, anthocyanins, and unique aroma compounds, strawberry fruit is highly favored by consumers. Fruit development and ripening constitute a highly coordinated, multi-step physiological program encompassing cell expansion, cell wall remodeling, sugar–acid metabolism, aroma formation, and anthocyanin accumulation [1]. Among these, the increase in fruit size and color transition represents the most direct morphological indicators, directly dictating commercial value [2]. These processes are finely governed by internal and external cues, including temperature, water availability, and phytohormones, with the developmental interaction between hormonal and sugar signals occupying a central regulatory position.

Fruits are broadly categorized into climacteric and non-climacteric types based on whether a surge in ethylene production occurs during ripening. Strawberry ripening is largely independent of ethylene and relies instead on a complex, alternative hormonal network. Classic studies have established that auxin, particularly indole-3-acetic acid (IAA), plays a paramount role during early fruit development. Nitsch et al. discovered that high levels of IAA synthesized in achenes post-fertilization are transported to the receptacle, driving cell division and extreme expansion; achene removal arrests receptacle growth, whereas exogenous auxin application partially restores this process [3]. Given et al. further demonstrated that synthetic auxin delays anthocyanin accumulation, chlorophyll degradation, and fruit softening, indicating that while IAA promotes early cell expansion, it exerts a marked inhibitory effect on late-stage ripening [4]. Endogenous IAA peaks at the small green stage and rapidly declines thereafter, closely matching the physiological transition from fruit growth to ripening initiation [5].

Gibberellin (GA) acts synergistically with IAA to primarily promote longitudinal fruit elongation. In woodland strawberry (*Fragaria vesca*), it was revealed that early high levels of IAA and GA activate the ABA catabolic gene *FveCYP707A4a*, thereby suppressing ABA accumulation to ensure normal fruit expansion; as IAA and GA levels decline, the ABA biosynthetic feedback loop is activated, leading to a burst in ABA that initiates ripening [6]. Csukasi et al. also confirmed that GA levels remain elevated at the white stage, participating in the regulation of receptacle development [7]. In stark contrast to IAA, ABA functions as a master positive regulator of ripening. Downregulating the key ABA biosynthetic gene *FaNCED1* via virus-induced gene silencing (VIGS) significantly reduces endogenous ABA levels and blocks fruit coloration, a phenotype rescued by exogenous ABA application; conversely, silencing the putative ABA receptor *FaCHLH/ABAR* renders the fruit insensitive to ABA, establishing the essential role of ABA and its signaling pathway in promoting fruit coloration, anthocyanin accumulation, and ripening [8]. In addition to individual hormonal signaling, the physiological transition of the receptacle from growth to ripening involves intricate multi-hormonal crosstalk and metabolic interactions. While ABA acts as a primary trigger for non-climacteric fruit ripening, recent studies demonstrate that ABA operates putative synergistic ally with sucrose accumulation and exhibits dynamic feedback interplay with auxins from the achenes to regulate vascular nutrient transport and receptacle cell volume expansion [9,10]. Furthermore, key transcriptional networks regulated by ABA and secondary messengers coordinate the expression of cell wall-modifying enzymes (e.g., cellulases and pectate lyases) and anthocyanin biosynthesis pathway genes, thereby orchestrating firmness loss and color development in parallel [11]. Exogenous ABA application accelerates color transition, increases anthocyanin content, and enhances the activities of cell wall-degrading enzymes. Sucrose functions not only as a pivotal metabolite but also as a crucial signaling molecule in regulating strawberry ripening. Exogenous sucrose and its non-metabolizable analogs significantly induce endogenous ABA accumulation, thereby accelerating ripening and coloration; silencing the sucrose transporter gene *FaSUT1* leads to a concomitant decline in sucrose and ABA levels, arresting fruit ripening, whereas *FaSUT1* overexpression produces the opposite effect [12]. Luo et al. further demonstrated a strong synergistic effect between ABA and sucrose: individual or combined treatments significantly shorten the developmental cycle, upregulate the expression of ABA biosynthesis/signaling genes (e.g., *NCED*, *SnRK2*), sucrose metabolism genes, and anthocyanin biosynthetic genes (e.g., *CHS*, *CHI*, *F3H*), while promoting the activities of cell wall-degrading enzymes [13]. Jia et al. identified the ASR (Abscisic acid, Stress, Ripening) transcription factor as a critical node linking ABA and sucrose signaling to co-regulate the ripening gene network. Although the independent roles of IAA, ABA, and sucrose across different stages have been extensively documented, how their stage-specific interactive crosstalk orchestrates cell expansion and anthocyanin biosynthesis remains to be systematically elucidated [14]. High early IAA levels drive cell expansion and suppress ABA accumulation to prevent premature ripening; following the color break stage, a decrease in IAA accompanied by an increase in ABA and sucrose jointly triggers the ripening program, including anthocyanin synthesis and cell wall remodeling. Through transcriptomic and hormonal profiling, Gu et al. further confirmed that IAA is predominantly synthesized in achenes to inhibit ripening, whereas ABA primarily accumulates in the receptacle to promote ripening [15]. Recent work also highlighted that the antagonistic relationship between IAA and ABA is a decisive factor governing fruit size and ripening onset [16].

However, the stage-specific spatio-temporal effects of exogenous IAA, ABA, and sucrose across six milestone developmental stages—small green (SG), medium green (MG), large green (LG), white (WT), initial red (IR), and fully red (FR)—on cell morphology, endogenous hormone dynamics, color parameters, softening enzyme activities, and key metabolic gene expression have not been fully clarified. Therefore, using the strawberry cultivar ‘Sweet Charlie’ as experimental material, this study systematically evaluated the impacts of exogenous IAA, ABA, and sucrose applications at distinct developmental stages on fruit cell expansion, anthocyanin biosynthesis, endogenous hormonal balance, cell wall metabolism, and relevant gene expression networks. The primary objective is to unravel the molecular and physiological mechanisms of spatio-temporal crosstalk among IAA, ABA, and sucrose in controlling strawberry fruit development and ripening, thereby enriching the theoretical framework of non-climacteric fruit ripening and providing a scientific basis for the precise regulation of strawberry fruit quality.

## 2. Materials and Methods

### 2.1. Plant Materials

Strawberry plants (*Fragaria* × *ananassa* cv. ‘Sweet Charlie’) were cultivated in a greenhouse at the Experimental Farm of Jinhua University of Vocational Technology, Jinhua, Zhejiang Province, China, during the winter of 2023–2024. Fruit development was categorized into six distinct stages: small green (SG), medium green (MG), large green (LG), white (WT), initial red (IR), and fully red (FR), corresponding to 7, 14, 18, 20, 22, and 26 days after anthesis (DAA), respectively. Upon harvesting, achenes were removed, and receptacle tissues were cut into 0.3 cm×0.5 cm blocks, snap-frozen in liquid nitrogen, and stored at −80 °C for subsequent biochemical and molecular analyses.

### 2.2. Abscisic Acid, Sucrose, and Auxin Treatments

Flowers were tagged at anthesis, and ten uniform fruits were selected at each developmental stage for each treatment. Fruits remained attached to the plant throughout the treatment period and the subsequent 5-day incubation. Injections were performed with a 1 mL syringe inserted along the longitudinal axis of the fruit. The injection volume was adjusted according to fruit size (200 µL) until liquid just began to seep from the base of the fruit, ensuring uniform infiltration; fruits showing excessive mechanical damage were discarded. Fruits were injected with 50 µM ABA, 20 µM IAA, or 30 mM sucrose. These concentrations were selected based on preliminary experiments that demonstrated clear physiological responses in strawberry without overt toxicity. Sterile water was injected as the mock control. Fruits were harvested for analysis 5 days after treatment. It should be noted that fruits treated at a given stage continued to progress developmentally during the 5-day period.

### 2.3. Morphological Observation of Fruit Cells

Paraffin sectioning was conducted on collected samples to observe anatomical changes under a light microscope (Leica DM2500, Leica Microsystems GmbH, Wetzlar, Germany). Fruits at six developmental stages (SG, MG, LG, WT, IR, FR) were treated for 5 days with 50 $\mu \mathrm{mol}\cdot L^{-1}$ ABA, 20 $\mu \mathrm{mol}\cdot L^{-1}$ IAA, 30 $\mathrm{mmol}\cdot L^{-1}$ sucrose, or sterile water. After treatment, fruits were bisected along the maximum longitudinal diameter. The tissue sections were rapidly fixed in FAA fixative (containing 5% *v*/*v* formaldehyde, 5% *v*/*v* glacial acetic acid, and 90% *v*/*v* of 90% ethanol). Following paraffin embedding, 9 μm thick sections were cut, stained with Safranin O-Fast Green (1.0% *w*/*v* Safranin O, 0.5% *w*/*v* Fast Green), mounted with neutral balsam, and photographed using a Leica optical microscope [17].

### 2.4. Determination of Anthocyanin and Vitamin C Contents

Total anthocyanins were extracted with 20 mL of 1% (*v*/*v*) HCl-anhydrous methanol in the dark at room temperature for 12 h. The extract was adjusted to 50 mL with extraction solvent in an amber volumetric flask, and absorbance was recorded at 530 nm and 657 nm. Net absorbance was calculated as:

$$A=A_{530}-0.25\times A_{657}$$

to correct for chlorophyll interference. One unit of anthocyanin concentration was defined as A=0.01 [18].

Ascorbic acid (Vitamin C) content was quantified using reverse-phase high-performance liquid chromatography (RP-HPLC). Fresh juice was centrifuged at 2000× *g* for 5 min at 4 °C. A 0.5 mL aliquot of supernatant was vortexed for 5 min and centrifuged at 9000× *g* for 10 min at 4 °C. The clear supernatant was analyzed on an RP-HPLC system equipped with a UV detector (Gilson Model 802/302/232, Anachem, Bedfordshire, UK). Chromatographic parameters: 20 μL injection volume, 248 nm detection wavelength, $1.0\;\mathrm{mL}\cdot \min^{-1}$ flow rate, using a Nucleosil ODS column (250 mm×4.6 mm i.d., 5 μm; Jones Chromatography, Hengoed, UK) fitted with a C18 guard column (38–40 μm; Anachem). The mobile phase (pH 5.5) consisted of 25 mM tetradecyltrimethylammonium bromide, 0.05 M NaOH, 0.06 M acetic acid, and 7.5% (*v*/*v*) acetonitrile, supplemented with $100\;\mathrm{mg}\cdot L^{-1}$ homocysteine and $200\;\mathrm{mg}\cdot L^{-1}$ EDTA prior to use [19].

### 2.5. Color Parameters and Fruit Firmness

Fruit longitudinal and equatorial diameters were measured using a digital vernier caliper. Fruit surface color was recorded in the CIE $L^{*}a^{*}b^{*}$ color space using a colorimeter, where $L^{*}$ indicates lightness, $a^{*}$ represents red-green chromaticity, and $b^{*}$ represents yellow-blue chromaticity. Color parameters were calculated as follows:

- Hue angle: $h^{\circ }=\arctan\left(b^{*}/a^{*}\right)$
- Chroma: $C^{*}=\sqrt{a^{*2}+b^{*2}}$
- Color Index for Red Fruit: $\mathrm{CIRG}=\left(180^{\circ }-h^{\circ }\right)/\left(L^{*}+C^{*}\right)$

Fruit firmness was evaluated at two opposite equatorial points per fruit after removing a thin layer of skin (∼1 mm). Measurements were performed using a digital fruit firmness tester (penetrometer) equipped with a 3.5 mm diameter cylindrical probe. The probe was pressed into the pulp to a depth of 5 mm at a uniform speed, and peak force was recorded in $\mathrm{kg}\cdot {\mathrm{cm}}^{-2}$. Five individual fruits were measured per replicate [20].

### 2.6. Total Soluble Solids, Titratable Acidity, Soluble Sugars

Total soluble solids (TSS) were determined using a PAL-1 handheld digital refractometer (ATAGO Co., Ltd., Tokyo, Japan). Titratable acidity (TA) was measured by titration with $0.1\;\mathrm{mol}\cdot L^{-1}$ NaOH and expressed as tartaric acid percentage. Soluble sugar content was determined via the anthrone-sulfuric acid method: 0.5 g of fresh tissue (FW) was sliced, mixed with 5 mL of distilled water, and heated in a boiling water bath for 30 min. The extraction was repeated twice, supernatants were combined and adjusted to 10 mL, and absorbance was measured at 630 nm [21].

### 2.7. Determination of Soluble Sugar and Organic Acid Component Contents

Soluble sugars (glucose, fructose, and sucrose) and organic acids (citric, malic, and ascorbic acids) were extracted and quantified according to the methods of Milosavljević and Simkova with minor modifications [19,21]. Briefly, 1.0 g of frozen receptacle tissue was homogenized with 5 mL deionized water, shaken for 30 min at room temperature, and centrifuged at 10,000× *g* for 10 min at 4 °C. The supernatant was filtered through a 0.22 µm membrane. Sugars were separated on an NH_2_ column with acetonitrile–water (80:20, *v*/*v*) and detected by RID. Organic acids were separated on a C18 column with methanol–0.2% phosphoric acid (3:97, *v*/*v*) and detected at 210 nm.

### 2.8. Endogenous Phytohormone Quantification

Endogenous hormones (IAA, ABA, GAs) were quantified via ultra-performance liquid chromatography-tandem mass spectrometry (UPLC-MS/MS) [22,23]. Fresh fruit powder (0.2 g FW) was extracted with pre-cooled 80% (*v*/*v*) methanol (containing 0.1–1% formic acid or 0.4% acetic acid) overnight at 4 °C or via ultrasonication for 10–30 min. Following centrifugation (12,000× *g*, 4 °C, 15 min), supernatants were concentrated under nitrogen, reconstituted in acidic water, and purified through solid-phase extraction (SPE) cartridges (Oasis HLB or C18 Sep-Pak). Eluates were concentrated and filtered through 0.22 μm membranes before injection into a UPLC-MS/MS system equipped with a C18 column (2.1×50 mm, 1.7–1.8 μm). Separation was achieved with a 0.1% formic acid in water (A) and 0.1% formic acid in acetonitrile (B) gradient. Mass spectrometry operated in ESI positive (IAA, GAs) and negative (ABA) multiple reaction monitoring (MRM) modes. Stable isotope-labeled internal standards ([C136]-IAA, [H26]-ABA, deuterated GAs) were used for quantification ($\mathrm{ng}\cdot g^{-1}\;\mathrm{FW}$). Each stage included 3–5 biological replicates.

### 2.9. Cell Wall Metabolizing Enzyme Assays

Activities of polygalacturonase (PG), pectin methylesterase (PME), and cellulase (endo-1,4-β-glucanase, EGase) were assayed spectrophotometrically.

Enzyme Extraction: Tissue powder (1.0–2.0 g FW) was extracted at 4 °C in $50\;\mathrm{mmol}\cdot L^{-1}$ sodium acetate buffer (pH 5.0–6.0) containing $1\;\mathrm{mol}\cdot L^{-1}$ NaCl and 1–2% PVPP. Homogenates were centrifuged (12,000× *g*, 4 °C, 20–30 min) to obtain crude enzyme extracts.

PG Activity: Determined by measuring reducing sugar release using 3,5-dinitrosalicylic acid (DNS). Extracts were incubated with 0.3–0.5% (*w*/*v*) polygalacturonic acid (PGA) in $50\;\mathrm{mmol}\cdot L^{-1}$ sodium acetate buffer (pH 4.5–5.0) at 37 °C for 30–120 min. DNS reagent was added, boiled for 5–10 min, and absorbance measured at 540 nm against a galacturonic acid standard. Activity was expressed as $\mu g\;\mathrm{galacturonic}\;\mathrm{acid}\cdot g^{-1}\;\mathrm{FW}\cdot h^{-1}$ [20].

PME Activity: The 1.0 mL reaction system contained 0.1% (*w*/*v*) citrus pectin, 0.0015% (*w*/*v*) bromothymol blue, $3\;\mathrm{mmol}\cdot L^{-1}$ potassium phosphate buffer (pH 7.5), and crude extract. Absorbance decay at 620 nm was monitored at 37 °C. Activity was expressed as $\mathrm{nmol}\;\mathrm{galacturonic}\;\mathrm{acid}\cdot g^{-1}\;\mathrm{FW}\cdot \min^{-1}$ [24].

Cellulase Activity: Determined using carboxymethyl cellulose (CMC) substrate via DNS reducing sugar assay at 540 nm against a glucose standard. One unit was defined as the amount of enzyme releasing 1μg glucose per gram FW per unit time [25].

### 2.10. RNA Extraction and qRT-PCR Analysis

Total RNA was extracted using the CTAB method and treated with RNase-free DNase I (TaKaRa, Kusatsu, Japan). RNA concentration and purity were verified using a One Drop-1000+ spectrophotometer, and structural integrity was checked by 1.0% agarose gel electrophoresis. cDNA synthesis was performed using a PrimeScript RT Reagent Kit (TaKaRa).

Quantitative real-time PCR (qRT-PCR) was executed on a Bio-Rad iQ2 Real-Time PCR system using SYBR Premix Ex Taq (TaKaRa) with FaACTIN as the internal reference. FaACTIN was selected because it has been validated as a stably expressed reference gene across strawberry fruit developmental stages in multiple independent studies [26,27]. The 20 μL reaction contained 1.0 μL cDNA template, 0.8 μL forward/reverse primers (10 μM each), 10 μL SYBR Premix, and 7.4 μL ${\mathrm{ddH}}_{2}O$. Thermal cycling: initial denaturation at 95 °C for 1 min, followed by 40 cycles of 95 °C for 10 s, annealing at 58 °C for 20 s, and extension at 72 °C for 30 s. Melting curves were analyzed post-amplification. Relative gene expression was calculated using the $2^{-\Delta \Delta C_{T}}$ method across three technical replicates per sample. See Table S1 for the primer sequences.

### 2.11. Statistical Analysis

Data are presented as mean ± SD. For experiments involving more than two groups, differences among treatments or developmental stages were analyzed by one-way ANOVA followed by Duncan’s multiple range test using SPSS 26.0, with *p* < 0.05 considered significant. For Table 1, each treatment was compared directly with the untreated control using a two-sided unpaired Student’s *t*-test; significance was defined as * *p* ≤ 0.05 and ** *p* ≤ 0.01. The number of biological replicates is stated in the corresponding figure or table legend. Technical qRT-PCR replicates were averaged before statistical analysis.

## 3. Results

### 3.1. Determination of Physiological and Quality Parameters During Strawberry Fruit Development

**Figure 1:**
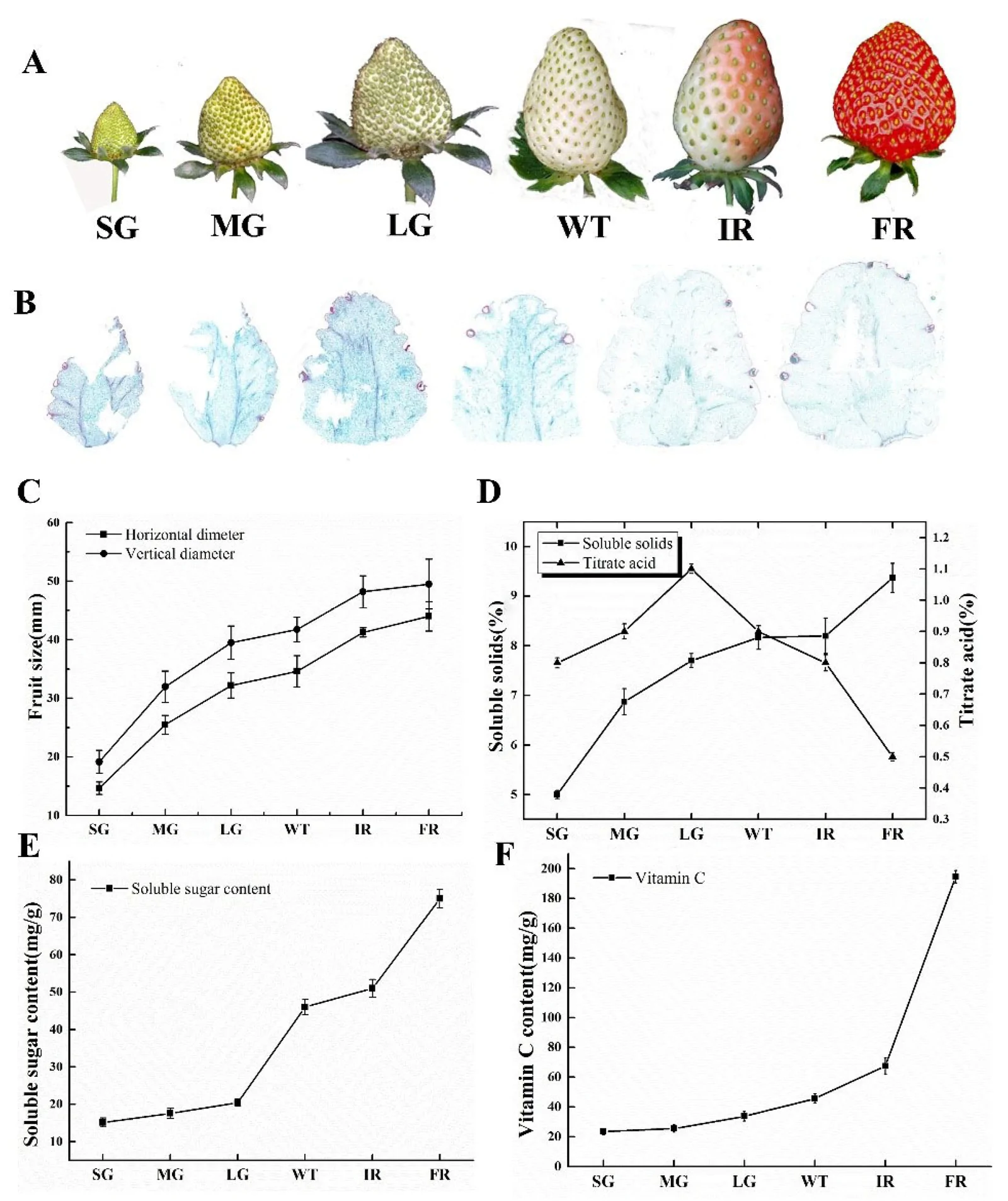
Developmental stages and quality parameters of ‘Sweet Charlie’ strawberry fruit. (**A**) Visual appearance of fruits at six developmental stages: small green (SG), medium green (MG), large green (LG), white (WT), initial red (IR) and full red (FR). (**B**) Cell structure of fruits at six developmental stages: small green (SG), medium green (MG), large green (LG), white (WT), initial red (IR) and full red (FR). (**C**) Changes in fruit longitudinal and transverse diameters. (**D**) Soluble solid content and titratable acidity. (**E**) Soluble sugar content. (**F**) Vitamin C content. Data are means ± SD (n = 3).

Based on fruit size and coloration, the developmental process of strawberry fruit was divided into six distinct stages: small green (SG), medium green (MG), large green (LG), white (WT), initial red (IR), and full red (FR) (Figure 1A). Cross-sections of the receptacle (Figure 1B) illustrate progressive tissue expansion and the gradual formation of larger intercellular spaces as development advanced.

Fruit size increased continuously throughout development (Figure 1C). Both horizontal and vertical diameters showed rapid growth from the MG to LG stage and continued expansion from the WT to FR stage, reflecting active cell expansion during these periods.

Soluble solids content (SSC) exhibited an overall upward trend and reached its maximum of 9.37% at the FR stage (Figure 1D). A relatively rapid increase in SSC occurred from the SG to MG stage and from the IR to FR stage. In contrast, titratable acidity (TA) increased during early development, peaked at the LG stage, and declined thereafter. The early accumulation of organic acids likely contributed to the elevated TA at the LG stage, while intensified metabolic activity during later ripening led to the progressive decrease in TA.

Soluble sugar content increased continuously throughout fruit development (Figure 1E). A pronounced rise was observed from the LG to WT stage and from the IR to FR stage, with the highest level recorded at the FR stage. Similarly, vitamin C content increased progressively (Figure 1F). From the SG to IR stages the increase was gradual, followed by a sharp rise from the IR to FR stage, reaching its maximum at full maturity.

Collectively, these results indicate that strawberry fruit undergoes two major phases of rapid growth (MG–LG and WT–IR), accompanied by progressive accumulation of soluble solids, sugars, and vitamin C, while titratable acidity peaks at the large-green stage and declines during ripening. These physiological changes lay the foundation for the subsequent hormonal and metabolic transitions that drive ripening.

### 3.2. Effects of Sucrose, Abscisic Acid, and IAA on Fruit Development and Cellular Changes at Different Developmental Stages

**Figure 2:**
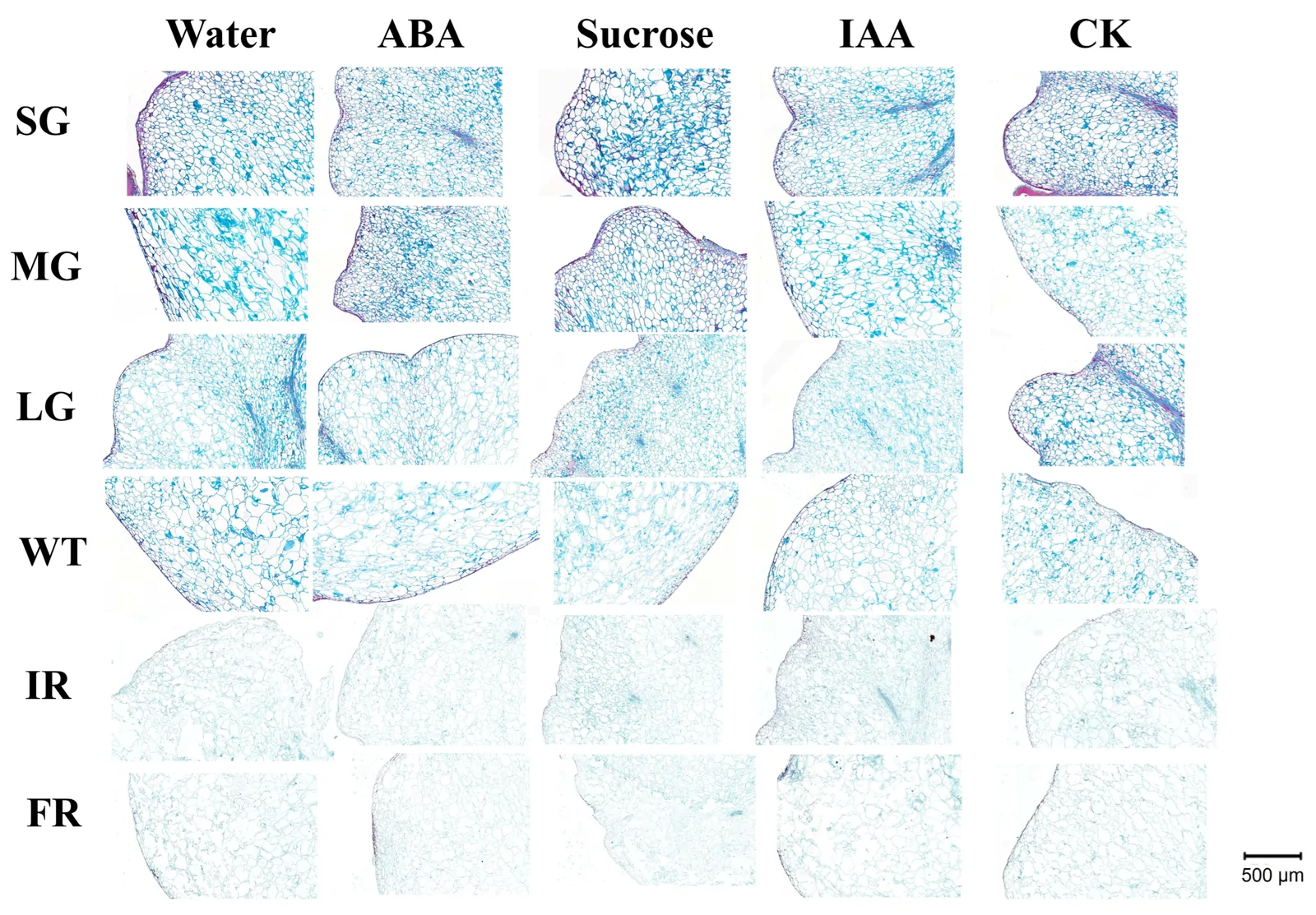
Effects of exogenous ABA, sucrose and IAA treatments on cellular morphology of strawberry fruit at different developmental stages. Paraffin sections of fruits treated at SG, MG, LG, WT, IR and FR stages and sampled 5 days later. CK, untreated control; Water, water control; ABA, 50 μM ABA; Sucrose, 30 mM sucrose; IAA, 20 μM IAA. Scale bars are indicated in the images.

To investigate the effects of exogenous ABA, sucrose, and IAA treatments on the tissue structure of strawberry fruits at different developmental stages, paraffin sections were prepared and examined five days after treatment (Figure 2). The results showed obvious differences among treatments in terms of cellular morphology, intercellular spaces, and tissue compactness, and these differences became increasingly pronounced as fruit development progressed. During the green fruit stages (SG, MG, and LG), the cells of fruits treated with the control (CK) and water were relatively orderly arranged, with clearly defined cell walls and relatively small intercellular spaces. Following ABA and sucrose treatments, some regions exhibited increased cell size and slightly enlarged intercellular spaces, indicating the beginning of tissue loosening. In contrast, IAA-treated fruits exhibited more compactly arranged cells with relatively regular cellular morphology, and the overall tissue maintained a higher degree of compactness. Upon entering the white fruit stage (WT), the differences among treatments became more pronounced. Fruits treated with ABA and sucrose appeared to exhibit enlarged intercellular spaces, lighter cell wall staining, and localized cell enlargement or slight tissue disintegration. The degree of tissue loosening appeared greater than that observed in the control. In contrast, IAA-treated fruits maintained relatively compact cellular organization and smaller intercellular spaces, indicating that IAA inhibited tissue loosening.

At the initial red (IR) and full red (FR) stages, fruits in the CK and water treatments exhibited typical characteristics of fruit maturation, including enlarged intercellular spaces, partial cell wall degradation, and increased tissue loosening. ABA and sucrose treatments further enhanced these changes, resulting in larger intercellular spaces, more irregular cell morphology, and a higher degree of tissue disintegration, indicating accelerated fruit maturation and softening. In contrast, cells in IAA-treated fruits remained relatively compactly arranged, with smaller intercellular spaces and greater tissue integrity, suggesting that IAA was associated with delayed tissue loosening and fruit softening. Overall, exogenous ABA and sucrose treatments were associated with the enlargement of intercellular spaces and tissue loosening during the later stages of strawberry fruit development, particularly from the WT to FR stages. These changes were consistent with their physiological effects in promoting fruit coloration and softening. In contrast, IAA treatment maintained relatively compact cellular organization and inhibited tissue loosening throughout fruit development, consistent with its role in delaying fruit maturation. The differences between the water and CK treatments were relatively minor, indicating that the solvent itself had limited effects on tissue structure. Collectively, these histological observations provide cellular-level evidence for the promotive effects of ABA and sucrose and the inhibitory effect of IAA on strawberry fruit maturation.

### 3.3. Effects of Sucrose, ABA, and IAA on Strawberry Fruit Coloration

**Figure 3:**
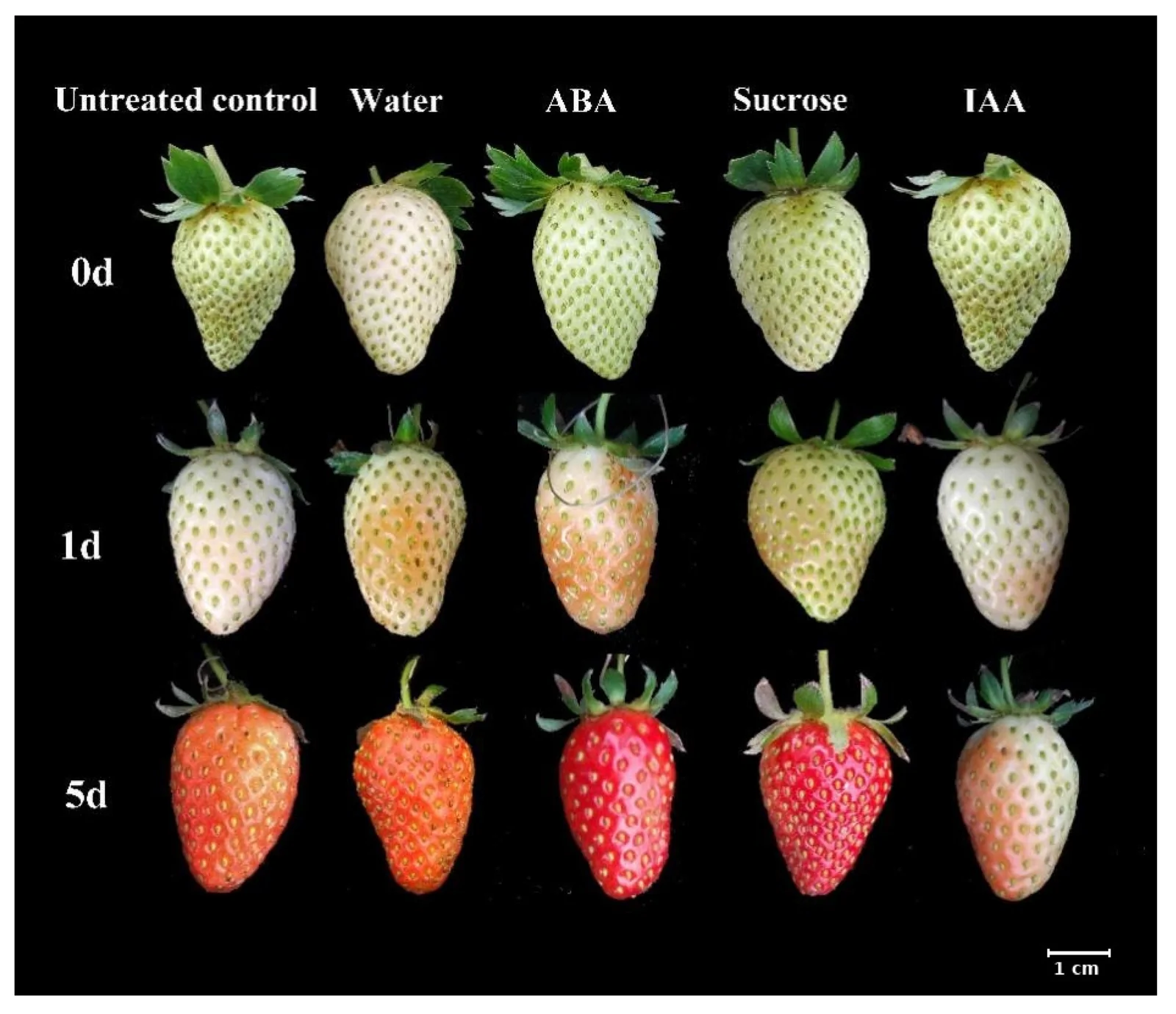
Effects of exogenous ABA, sucrose and IAA on fruit coloration and anthocyanin accumulation in white-stage (WT) strawberry fruit. Fruits were treated at the WT stage and analyzed 5 days later.

To determine the effects of exogenous ABA, sucrose, and IAA on strawberry fruit coloration at the white fruit stage (WT), anthocyanin content and color parameters (L, a*, b*, C, H, and CIRG) were measured five days after treatment (Table 1). Compared with the untreated control, treatments with ABA (50 μM) and sucrose (30 mM) significantly promoted fruit coloration. The highest anthocyanin content was detected in ABA-treated fruits, reaching 161.72 mg/kg, approximately threefold that of the untreated control, followed by sucrose-treated fruits, which reached 119.98 mg/kg, approximately 2.3-fold that of the control. Consistent with the increase in anthocyanin accumulation, the L value (lightness) decreased markedly following ABA and sucrose treatments, reaching 32.71 and 34.09, respectively, indicating a darker fruit color and reduced lightness. Although the a* value showed some variation, the CIRG value increased significantly to 2.28 and 2.37 following ABA and sucrose treatments, respectively, indicating a pronounced increase in fruit redness and an accelerated progression of coloration and ripening. Water treatment resulted in a slightly higher anthocyanin content (78.72 mg/kg) and CIRG value (1.80) than the untreated control; however, these changes were much smaller than those induced by ABA and sucrose, indicating that the solvent itself had only a limited effect on fruit coloration. In contrast, IAA (20 μM) treatment significantly inhibited fruit coloration (Figure 3). The anthocyanin content was the lowest in IAA-treated fruits, reaching only 20.75 mg/kg, approximately 39% of that in the untreated control. The L value increased significantly to 56.65, indicating that the fruits retained higher lightness and a relatively pale color. Meanwhile, the H value increased to 49.28, whereas the CIRG value decreased to 1.49, further indicating that red color development was suppressed and fruit ripening was markedly delayed by IAA treatment.

Overall, exogenous ABA and sucrose effectively promoted the transition from white to red coloration and accelerated strawberry fruit ripening, as evidenced by significantly enhanced anthocyanin accumulation, reduced lightness, and increased CIRG values. In contrast, IAA treatment markedly delayed fruit coloration by suppressing anthocyanin accumulation while maintaining higher lightness and a lower CIRG value. The changes in color parameters were highly consistent with the variation in anthocyanin content, providing complementary visual and biochemical evidence for the promotive effects of ABA and sucrose and the inhibitory effect of IAA on strawberry fruit ripening.

### 3.4. Effects of Sucrose, ABA, and IAA Treatments on Endogenous Hormone Levels in Strawberry Fruits

**Figure 4:**
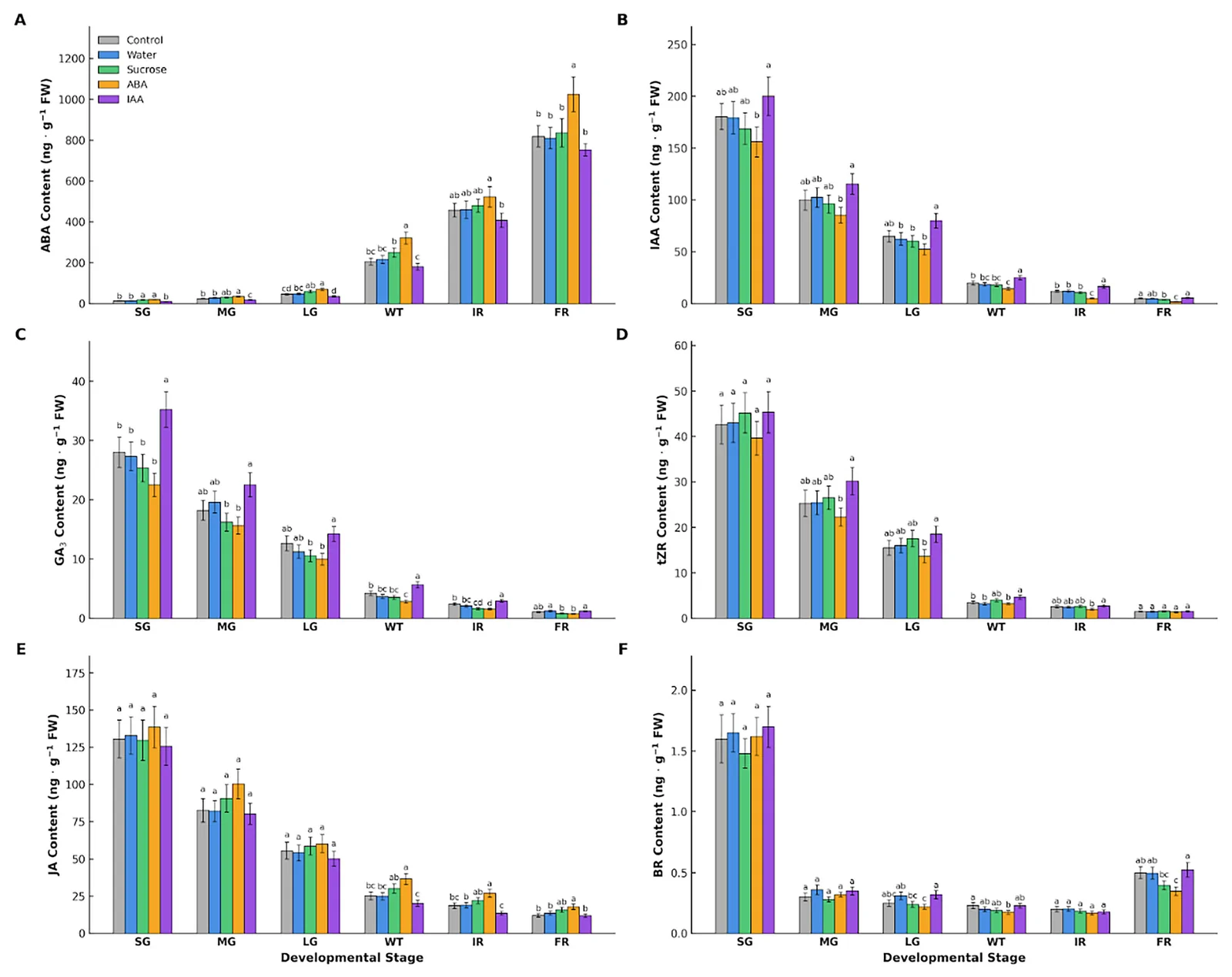
Dynamic changes in endogenous hormone contents in strawberry fruit under different treatments across developmental stages. Contents of ABA (**A**), IAA (**B**), GA_3_ (**C**), tZR (**D**), JA (**E**), and BR (**F**) in fruits treated with water, ABA, sucrose or IAA at SG to FR stages. Values are means ± SD (Standard deviation) of five biological replications. Different lowercase letters above the bars indicate significant differences among treatments or developmental stages at *p* < 0.05, as determined by one-way analysis of variance (ANOVA) followed by Duncan’s multiple range test.

Exogenous treatments significantly altered the dynamic balance of endogenous hormones during strawberry fruit development, with distinct stage-specific patterns observed across different developmental stages. During the early stages of fruit development, when cell division and tissue growth were particularly active (SG to LG), IAA, GA_3_, tZR, JA, and BR were maintained at relatively high levels (Figure 4B–F). IAA reached its maximum level at the SG stage, while GA_3_ and tZR were also maintained at relatively high levels, suggesting that these growth-promoting hormones may act coordinately to regulate cell division and organ development during the early stages of fruit development. As fruit development progressed, the levels of these hormones declined sharply from the white fruit stage (WT) to the full-red stage (FR).

In contrast, endogenous ABA remained at a very low level during the early developmental stages (SG to LG), with concentrations below 100 ng·g^−1^ FW but increased dramatically during the coloration and ripening stages (WT to FR), reaching its highest level at the FR stage(Figure 4A). Exogenous treatments significantly modulated this physiological transition. Exogenous ABA treatment markedly promoted endogenous ABA accumulation through a positive feedback mechanism, thereby accelerating fruit ripening. Exogenous IAA treatment effectively maintained the auxin pool and concomitant GA_3_ accumulation during the early fruit developmental stages. In addition, exogenous sucrose and ABA treatments positively regulated the levels of JA and other hormones associated with defense responses and fruit ripening at specific developmental stages. These results highlight the complex cross-talk among exogenous signaling molecules and endogenous hormone pathways and suggest that such interactions may contribute to the coordinated regulation of sugar metabolism, fruit maturation, and senescence in strawberry.

### 3.5. Effects of Sucrose, ABA, and IAA Treatments on Fruit Softening-Related Enzymes and Gene Expression in Strawberry Fruits

Fruit softening is an inevitable physiological process accompanying strawberry fruit maturation. To investigate the effects of sucrose, ABA, and IAA on fruit softening at different developmental stages, strawberry fruits at different developmental stages were subjected to the respective treatments and sampled five days later for determination of fruit firmness and the activities of cell wall metabolism-related enzymes, including polygalacturonase (PG), pectin lyase (PL), pectin methylesterase (PME), and cellulase. The results showed that the effects of exogenous treatments on fruit softening varied considerably depending on the developmental stage.

**Figure 5:**
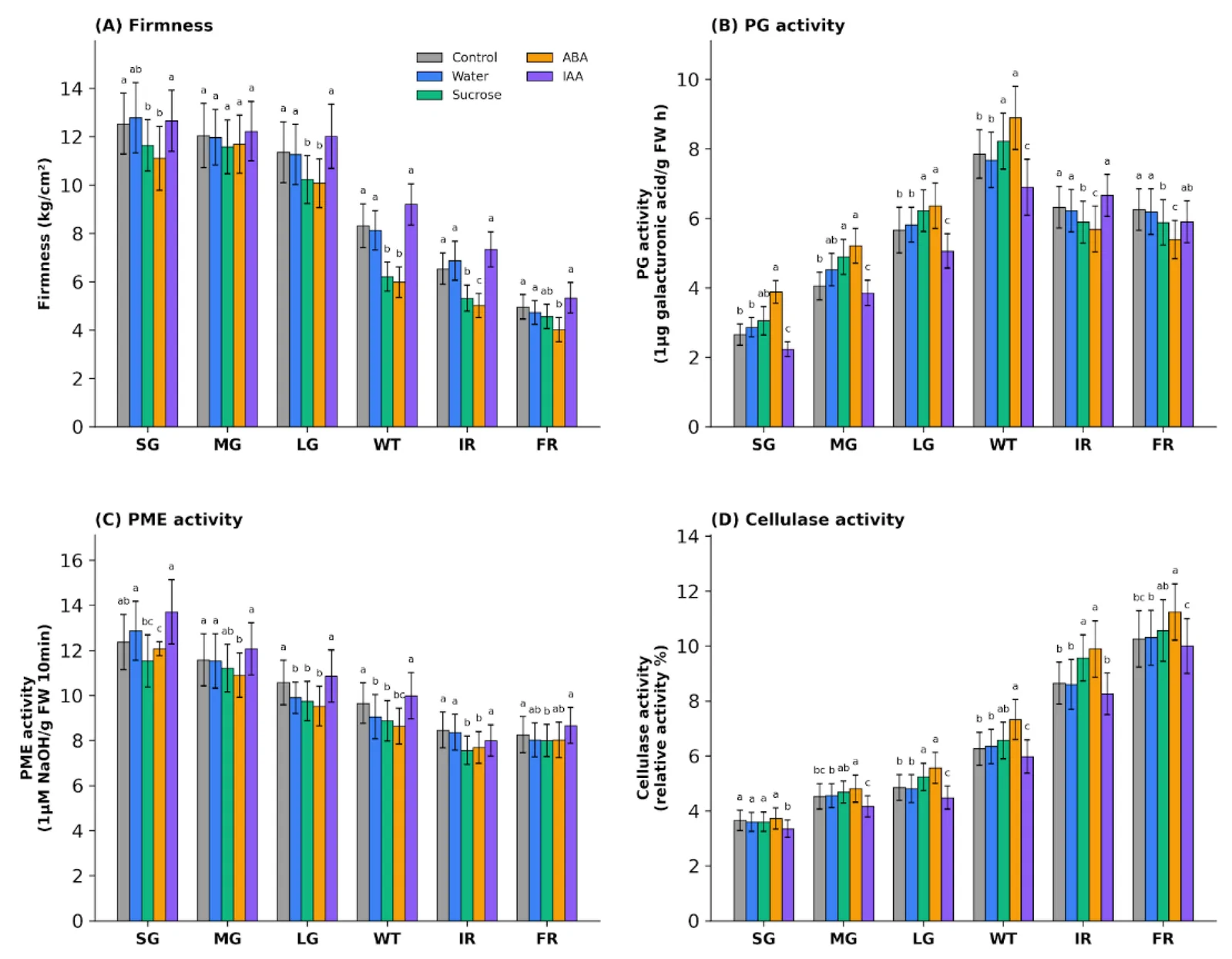
Effects of exogenous ABA, sucrose and IAA on fruit firmness and cell-wall degrading enzyme activities during strawberry fruit development. (**A**) Fruit firmness. (**B**) Polygalacturonase (PG) activity. (**C**) Pectin methylesterase (PME) activity. (**D**) Cellulase activity. Fruits were treated at each developmental stage and sampled 5 days later. Values are means ± SD (Standard deviation) of five biological replications. Different lowercase letters above the bars indicate significant differences among treatments or developmental stages at *p* < 0.05, as determined by one-way analysis of variance (ANOVA) followed by Duncan’s multiple range test.

Exogenous treatments significantly affected cell wall metabolism and fruit textural properties during strawberry fruit development and ripening. As fruit development progressed from the green stages (SG, MG, and LG) to the coloration and ripening stages (WT, IR, and FR), fruit firmness continuously decreased (Figure 5A). In the control group, firmness decreased significantly from 12.54 kg/cm^−2^ at the early developmental stages to 4.96 kg/cm^−2^ at the FR stage. This progressive loss of firmness was accompanied by pronounced changes in the activities of cell wall-degrading enzymes (Figure 5A). PG activity increased continuously throughout fruit development and ripening, increasing from 2.65 to 6.25 μg galacturonic acid g^−1^ FW h^−1^ (Figure 5B). Similarly, the activities of PME, PL, and cellulase exhibited dynamic changes that were closely associated with the progression of fruit softening (Figure 5C,D).

The different exogenous treatments exerted distinct effects on these physiological processes. Exogenous ABA and sucrose treatments significantly accelerated the loss of fruit firmness, which was accompanied by marked increases in the activities of cell wall-degrading enzymes, particularly PG. In contrast, exogenous IAA treatment partially maintained higher fruit firmness and suppressed the activities of cell wall-degrading enzymes. These results indicate that exogenous signaling molecules can differentially regulate strawberry fruit softening by modulating the activities of key cell wall-degrading enzymes, such as PG and cellulase, thereby promoting or delaying the depolymerization of cell wall polysaccharides and consequently regulating the progression of fruit maturation and softening.

### 3.6. Effects of Sucrose, ABA, and IAA Treatments on the Dynamic Accumulation of Sugars and Organic Acids in Strawberry Fruits

Exogenous treatments exerted significant stage-specific effects on the dynamic accumulation of sugars and organic acids during strawberry fruit development and ripening. Overall, the major soluble sugars, including glucose, fructose, and sucrose, as well as malic acid, showed pronounced accumulation during the later stages of fruit development. In contrast, citric acid and isocitric acid exhibited relatively stable fluctuations throughout fruit development.

**Figure 6:**
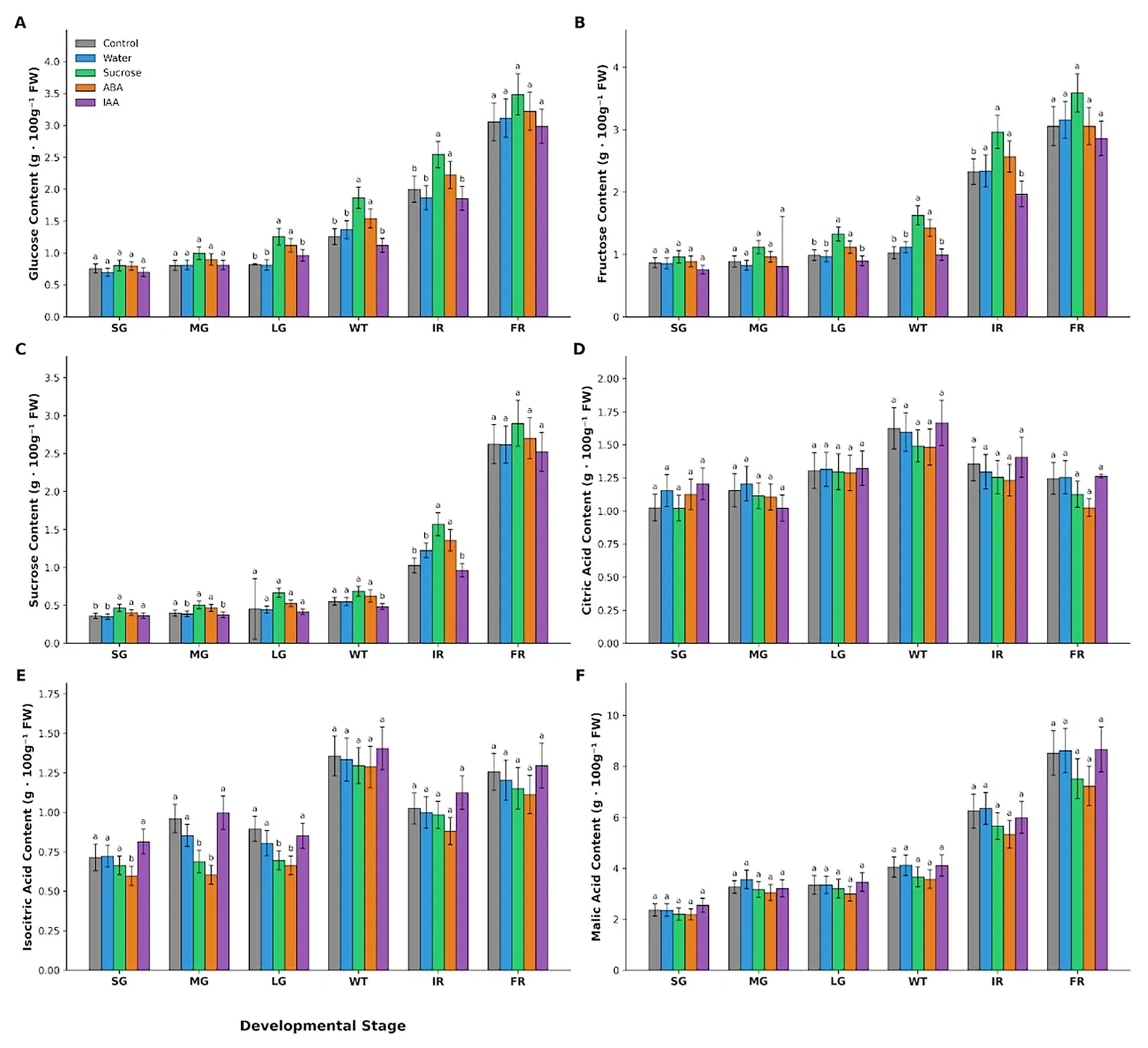
Effects of exogenous ABA, sucrose and IAA on soluble sugar and organic acid components in strawberry fruit. Contents of (**A**) glucose, (**B**) fructose, (**C**) sucrose, (**D**) citric acid, (**E**) isocitric acid, (**F**) malic acid and at different developmental stages under the indicated treatments. Values are means ± SD (Standard deviation) of five biological replications. Different lowercase letters above the bars indicate significant differences among treatments or developmental stages at *p* < 0.05, as determined by one-way analysis of variance (ANOVA) followed by Duncan’s multiple range test.

During the early developmental stages (SG to LG), the contents of glucose (Figure 6A), fructose (Figure 6B), and sucrose (Figure 6C) remained relatively low. Following the transition to the white fruit stage (WT), the levels of all three soluble sugars increased rapidly and reached their maximum levels at the full-red stage (FR), with both glucose and fructose exceeding 3.0 g 100 g^−1^ FW. Exogenous sucrose and ABA treatments markedly promoted sugar accumulation, particularly during the WT to IR stages. The contents of glucose, fructose, and sucrose in sucrose-treated fruits were significantly higher than those in the control group (*p* < 0.05), followed by the ABA treatment. In contrast, exogenous IAA treatment partially suppressed the accumulation of soluble sugars, with sugar contents generally lower than those of the control during the ripening stages.

Among the organic acids, malic acid (Figure 6F), the major organic acid component, showed a continuous increasing trend during fruit development and reached its highest level at the FR stage. Exogenous sucrose and ABA treatments maintained relatively lower malic acid levels during the later stages of ripening, which may contribute to an increase in the sugar-to-acid ratio. In contrast, citric acid (Figure 6D) and isocitric acid (Figure 6E) exhibited relatively modest fluctuations throughout fruit development, with slight peaks occurring at the WT and FR stages, respectively.

Overall, exogenous ABA and sucrose treatments primarily promoted the accumulation of glucose, fructose, and sucrose during the coloration and ripening stages and coordinated the changes in organic acid levels during fruit maturation, thereby contributing to the formation of fruit quality characteristics during ripening. In contrast, exogenous IAA treatment tended to maintain metabolic characteristics associated with the early developmental stages and delayed the rapid accumulation of soluble sugars during fruit maturation.

### 3.7. Effects of Sucrose, ABA, and IAA Treatments on the Expression of Key Genes Involved in Major Metabolic Pathways

#### 3.7.1. Anthocyanin Biosynthetic Pathway

**Figure 7:**
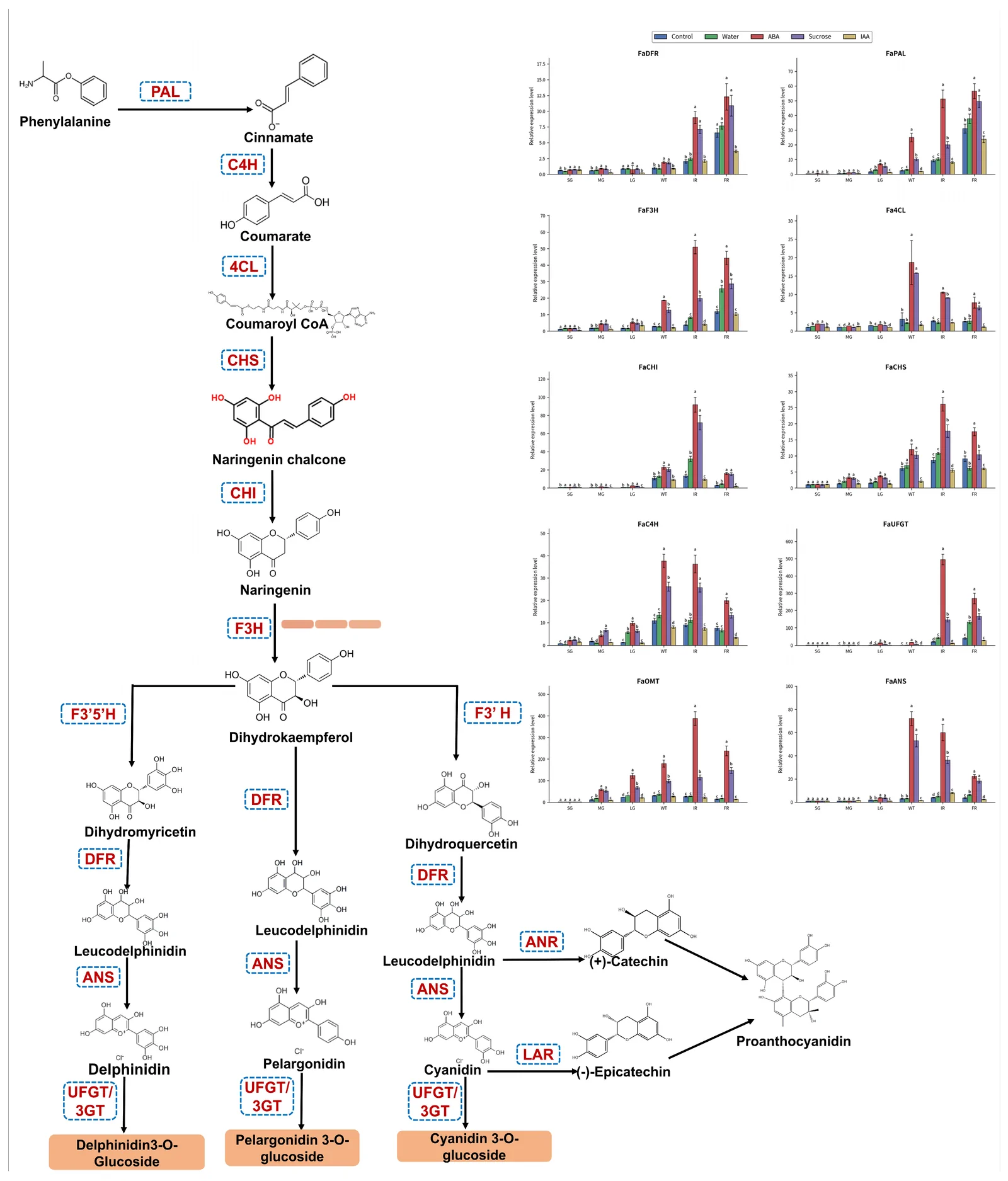
Expression profiles of key anthocyanin biosynthetic genes in strawberry fruit under different treatments. Relative expression levels of *FaDFR*, *FaPAL*, *FaF3H*, *Fa4CL*, *FaCHI*, *FaCHS*, *FaC4H*, *FaUFGT*, *FaOMT* and *FaANS* at SG, MG, LG, WT, IR and FR stages under Control, Water, ABA, Sucrose and IAA treatments. Different lowercase letters above the bars indicate significant differences among treatments or developmental stages at *p* < 0.05, as determined by one-way analysis of variance (ANOVA) followed by Duncan’s multiple range test.

The expression profiles of 10 key anthocyanin biosynthetic genes (*FaDFR*, *FaPAL*, *FaF3H*, *Fa4CL*, *FaCHI*, *FaCHS*, *FaC4H*, *FaUFGT*, *FaOMT* and *FaANS*) exhibited clear stage-specific and treatment-dependent patterns (Figure 7). During the early developmental stages (SG–LG), most genes remained strongly repressed, as indicated by the predominantly dark-blue signals in the heatmap. Upon entering the white stage (WT), the pathway was broadly activated, and transcript levels of the majority of structural genes rose markedly during the IR and FR stages, reaching their highest abundance (dark-red signals). This temporal pattern was highly consistent with the rapid accumulation of anthocyanins observed during fruit coloration. Exogenous ABA and sucrose treatments substantially elevated the expression of key structural genes (*FaDFR*, *FaF3H*, *FaUFGT* and *FaANS*), particularly at the IR and FR stages, and advanced the activation of FaC4H, *FaOMT* and *FaANS* as early as the WT stage. Notably, *FaUFGT* and *FaOMT* showed especially strong induction under ABA treatment. In contrast, IAA treatment markedly attenuated the transcriptional induction of most target genes during the WT and IR stages; even at the FR stage, genes such as *FaDFR*, *FaF3H* and *FaCHI* maintained relatively low expression levels. Collectively, these results demonstrate that ABA and sucrose promote the coordinated transcriptional activation of the anthocyanin biosynthetic pathway, whereas IAA delays its activation, thereby supporting the differential regulatory roles of these signals in strawberry fruit coloration.

#### 3.7.2. Sugar Metabolism in Strawberry Fruits

**Figure 8:**
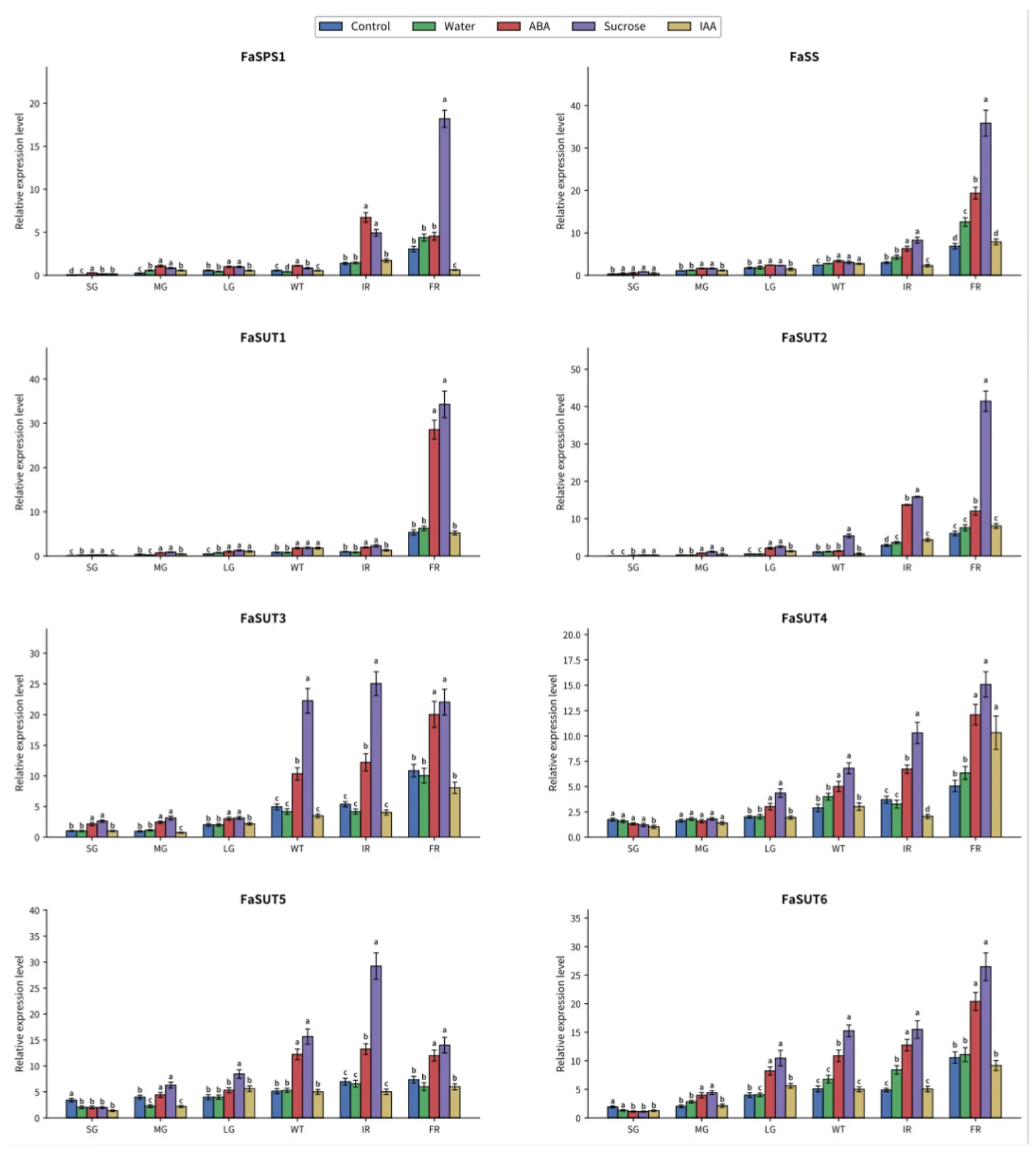
Expression profiles of sugar metabolism and transport genes in strawberry fruit under different treatments. Relative expression levels of *FaSPS1*, *FaSS*, *FaSUT1*, *FaSUT2*, *FaSUT3* and *FaSUT4* at six developmental stages under the indicated treatments. Different lowercase letters above the bars indicate significant differences among treatments or developmental stages at *p* < 0.05, as determined by one-way analysis of variance (ANOVA) followed by Duncan’s multiple range test.

The expression patterns of genes involved in sugar biosynthesis (*FaSPS1* and *FaSS*) and sugar transport (*FaSUT1*–*FaSUT4*) were closely coordinated with fruit maturation and were markedly modulated by exogenous treatments (Figure 8). All examined genes remained at relatively low transcript levels during the early developmental stages (SG–MG–LG) and were strongly upregulated as ripening progressed. *FaSS* and *FaSUT1* exhibited pronounced late-ripening-specific expression, reaching their maximum abundance at the FR stage, whereas *FaSPS1*, *FaSUT2*, *FaSUT3* and *FaSUT4* were activated earlier, showing marked increases from the WT or IR stages onward. ABA treatment advanced the expression peaks of *FaSPS1* and *FaSUT2* from the FR stage to the IR stage and strongly induced *FaSUT3* and *FaSUT4* during the WT and IR stages. Sucrose treatment produced a similar inductive effect, particularly enhancing the transcript levels of FaSUT3 and FaSUT4 in the early ripening phases. IAA treatment selectively promoted FaSPS1 expression at the IR stage and FaSUT4 at the FR stage. These results indicate that the transcriptional activation of sugar-metabolism and -transport genes is tightly linked to strawberry fruit maturation, and that exogenous ABA and sucrose signals promote sugar accumulation by advancing and intensifying the expression of key sucrose-biosynthesis and -transport genes, especially during the white-to-red transition.

#### 3.7.3. Expression Levels of Genes Related to Strawberry Fruit Ripening

**Figure 9:**
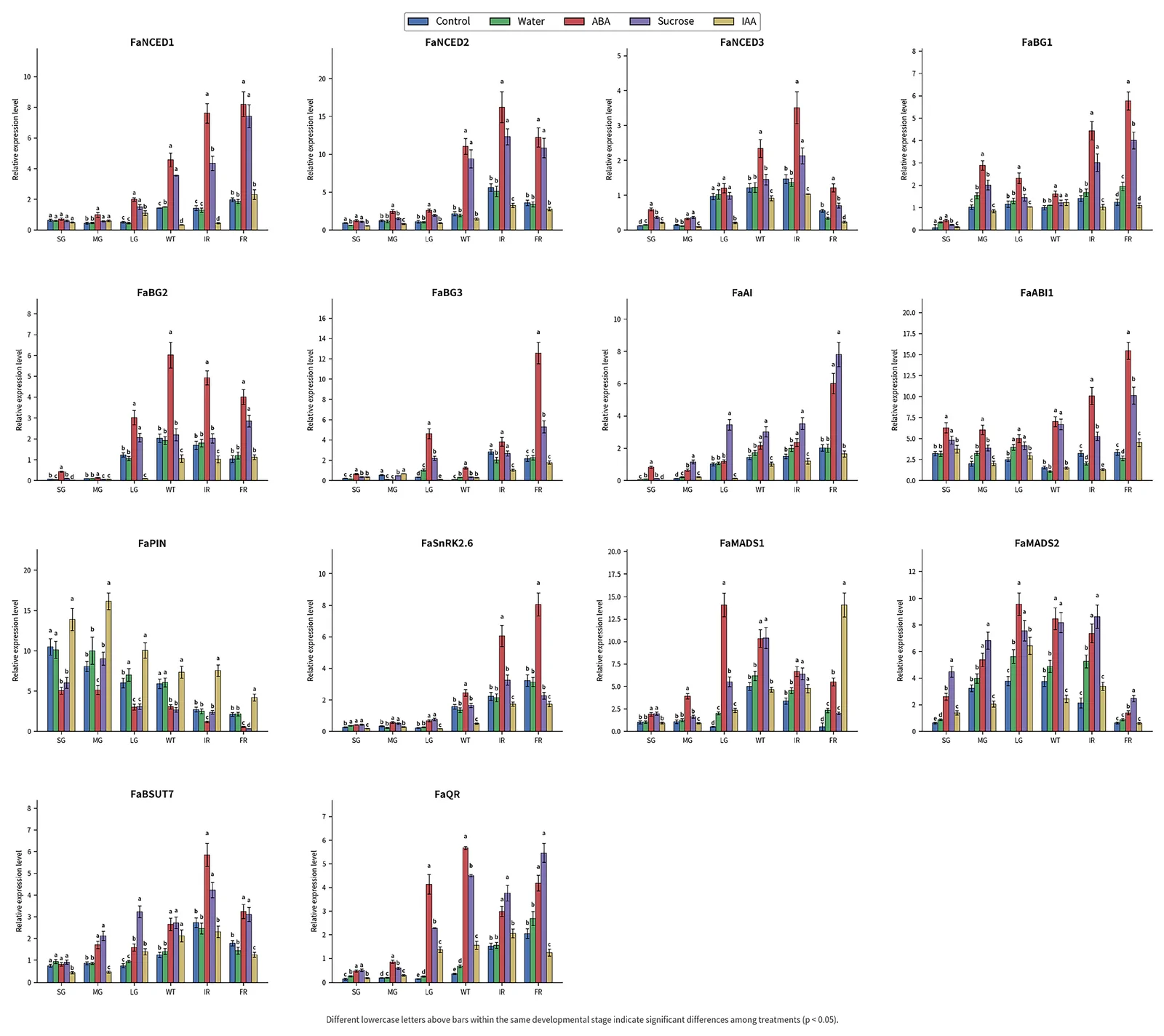
Expression profiles of ripening-related genes in strawberry fruit under different treatments. Relative expression levels of 14 candidate ripening-related genes (including *FaNCED1/2/3*, *FaBG1/3*, *FaAI*, *FaABI1*, *FaSnRK2.6*, *FaMADS1/2*, *FaPIN*, *FaBSUT7*, *FaQR*, etc.) at six developmental stages under Control, Water, ABA, Sucrose and IAA treatments. Different lowercase letters above the bars indicate significant differences among treatments or developmental stages at *p* < 0.05, as determined by one-way analysis of variance (ANOVA) followed by Duncan’s multiple range test.

14 ripening-related genes revealed clear stage-specific expression patterns and differential responses to exogenous treatments (Figure 9). The majority of genes (*FaNCED1/2/3*, *FaBG1/3*, *FaAI*, *FaABI1*, *FaSnRK2.6*, *FaMADS1*, *FaBSUT7* and *FaQR*) were strongly induced from the WT stage onward and reached peak expression during the IR and FR stages, indicating their central involvement in ripening-associated processes such as softening, sugar accumulation, pigment metabolism and ABA signal transduction. ABA and sucrose treatments exerted robust positive inductive effects on most of these genes; expression levels of FaNCED1, FaNCED2, FaABI1 and FaMADS1 under ABA or sucrose treatment were markedly higher than those in the Control and Water groups at the WT, IR and FR stages. In contrast, a minority of genes (*FaPIN* and *FaMADS2*) displayed higher expression during the green stages (SG–MG) and were progressively downregulated as ripening advanced; IAA treatment specifically modulated FaPIN at early stages while subsequently suppressing its expression during ripening. These results provide molecular evidence that ABA and sucrose signals synergistically accelerate the transcriptional activation of the ripening gene network, whereas IAA helps maintain the early developmental program and antagonizes the transition to ripening. Regarding exogenous treatments, ABA and sucrose exerted strong positive inductive effects on most ripening-related genes. For example, expression levels of *FaNCED1*, *FaNCED2*, *FaABI1*, and *FaMADS1* under ABA and sucrose treatments were markedly higher than those in the Control and Water groups during the WT, IR, and FR stages. These results provide compelling evidence that exogenous ABA and sugar signals can putative synergistic promote or accelerate the transcriptional activation of ripening-related genes, suggesting that sugar and ABA signaling pathways play central interactive roles in the regulatory network of fruit ripening [13,28,29].

## 4. Discussion

Strawberry fruit development is characterized by a highly coordinated transition from rapid receptacle growth to ripening-associated pigmentation, sugar accumulation and tissue softening. In the present study, ‘Sweet Charlie’ strawberry fruits exhibited rapid increases in fruit dimensions during the MG–LG and WT–IR transitions, whereas soluble solids and soluble sugars increased markedly during the later stages, particularly from IR to FR. These developmental changes were accompanied by a pronounced hormonal transition: IAA and other growth-associated hormones were relatively abundant during the early developmental stages, whereas ABA increased substantially from the white stage onward and reached its highest level at the fully red stage. This temporal separation strongly supports the concept that the balance between growth-promoting hormones and ABA acts as an important developmental switch between fruit expansion and ripening.

Nitsch demonstrated that removal of achenes inhibited receptacle enlargement, whereas exogenous auxin could partially restore growth, establishing auxin as a central signal linking fertilization to strawberry fruit development [3]. Subsequent studies demonstrated that auxin is primarily synthesized in the achenes and transported to the receptacle, where it promotes cell expansion and fruit growth [4,5]. More recent reporter-based analyses further showed that auxin biosynthesis is particularly active in the endosperm and embryo of strawberry achenes, providing spatial evidence for the achene-derived auxin model [30]. The decline in IAA observed during the transition toward ripening is also consistent with previous measurements of endogenous hormones in strawberry. Symons et al. showed that IAA increased rapidly during early fruit development but declined before the onset of intense color accumulation, whereas ABA increased progressively during development and ripening. Importantly, the present study not only reproduced this developmental pattern but also demonstrated that exogenous IAA maintained relatively high endogenous IAA levels and delayed several physiological events associated with ripening [5]. Thus, the inhibitory effect of IAA observed in the present study is unlikely to represent a simple effect of exogenous hormone application but rather reflects the developmental role of IAA in maintaining the pre-ripening physiological state.

ABA remained at a relatively low level during SG–LG but increased sharply from WT to FR, while IAA exhibited the opposite tendency. Previous genetic evidence demonstrated that reduction of ABA biosynthesis through silencing of FaNCED1 results in reduced ABA accumulation and delayed strawberry coloration, whereas exogenous ABA can restore the ripening phenotype [8]. Likewise, Liao et al. demonstrated that auxin and GA suppress ABA accumulation during early fruit development by promoting ABA catabolism through FveCYP707A4a, whereas the subsequent decline in auxin and GA releases ABA biosynthesis and facilitates the onset of ripening [6]. Therefore, the present results support a model in which high IAA during early development maintains low ABA activity and sustains fruit growth, while declining IAA permits ABA accumulation and initiates the ripening program. AUXIN RESPONSE FACTOR 2 has been shown to repress the ABA biosynthetic gene FaNCED1, providing a direct molecular connection between auxin signaling and ABA biosynthesis in strawberry receptacles [31]. Although the present study did not directly examine FaARF2–FaNCED1 interactions, the reciprocal IAA and ABA profiles observed here are consistent with the existence of such an auxin-mediated repression mechanism. Collectively, these observations suggest that the IAA/ABA ratio, rather than the absolute concentration of either hormone alone, may represent an important developmental indicator controlling the transition from expansion to ripening. Endogenous profiling revealed dynamic fluctuations in jasmonic acid (JA) and brassinosteroids (BR), both of which are known to modulate fruit maturation, flavor development, and stress responses [32,33]. Additionally, polyamines (PAs) act as crucial anti-senescence regulators that counteract ABA/ethylene action to maintain cell wall stability during postharvest storage [34]. However, how jasmonic acid, brassinosteroids, and polyamine signaling pathways interact with the ABA/auxin balance to drive precise second messenger cascades—such as cytosolic Ca^2+^ oscillations, reactive oxygen species (ROS) bursts, and localized phosphoinositide signaling—during late pericarp and receptacle softening events remains incompletely understood [32,33,34]. Elucidating these second messenger cascades will be essential for unraveling the fine-scale physiological triggers of cell wall breakdown in non-climacteric fleshy fruits.

The most prominent phenotype induced by ABA treatment in the present study was accelerated fruit coloration. Exogenous ABA markedly increased anthocyanin accumulation and CIRG values while decreasing fruit brightness, whereas IAA treatment produced the opposite effects. These results are in strong agreement with previous functional studies establishing ABA as a central positive regulator of strawberry ripening. Jia et al. showed that suppression of *FaNCED1* reduced ABA levels and resulted in uncolored fruits, whereas exogenous ABA rescued the phenotype [8]. Similarly, ABA treatment enhanced anthocyanin biosynthetic enzyme activities and accelerated strawberry ripening [35]. Thus, the enhanced anthocyanin accumulation observed in the present study is consistent with the established function of ABA as a ripening-promoting signal rather than simply being a secondary consequence of advanced fruit development.

At the transcriptional level, *FaMYB10* provides an important mechanistic link between ABA signaling and anthocyanin biosynthesis. *FaMYB10* is a major positive regulator of flavonoid and anthocyanin biosynthesis in strawberry, and its expression is repressed by auxin but activated by ABA [36].Moreover, ABA-induced *FaMYB10* expression is associated with activation of downstream structural genes involved in anthocyanin biosynthesis, including *CHS*, *CHI*, *F3H*, *DFR*, *ANS* and *UFGT* [36,37]. Therefore, the strong induction of multiple anthocyanin biosynthetic genes by ABA in the present study may reflect activation of a broader transcriptional regulatory network rather than independent regulation of each structural gene.

The strong response of *FaUFGT* and *FaOMT* to ABA is also noteworthy. *UFGT* catalyzes the glycosylation of anthocyanidins, producing stable anthocyanin glycosides, whereas *OMT* contributes to methylation reactions that modify flavonoid compounds. The early induction of these genes in ABA-treated fruit may facilitate the rapid conversion and stabilization of anthocyanin metabolites during the transition from white to red fruit. This interpretation is consistent with the observation that anthocyanin accumulation and CIRG increased markedly following ABA treatment. Furthermore, light and ABA have both been reported to stimulate *FaMYB10* and anthocyanin accumulation, suggesting that environmental and hormonal signals may converge on common transcriptional regulators of strawberry pigmentation [37].

In contrast, IAA strongly suppressed the activation of anthocyanin-related genes, particularly during the WT and IR stages. This agrees with earlier observations that auxin delays strawberry coloration and represses ripening-associated genes [38,39,40]. Thus, the antagonistic effects of IAA and ABA on fruit pigmentation appear to operate at multiple levels, involving hormonal accumulation, ripening-associated transcription factors and downstream anthocyanin biosynthetic genes.

The role of sucrose in the present study is consistent with the landmark finding that sucrose acts as a signaling molecule during strawberry fruit ripening. Jia et al. demonstrated that exogenous sucrose and the non-metabolizable sucrose analogue turanose accelerated strawberry ripening and stimulated ABA accumulation [12]. More importantly, suppression of *FaSUT1* reduced sucrose and ABA levels and delayed ripening, whereas *FaSUT1* overexpression increased both sucrose and ABA and accelerated fruit ripening. These results indicate that sucrose is not merely a passive substrate accumulating as a consequence of maturation but can function upstream of ABA signaling. Sucrose treatment increased the expression of ripening-associated *FaNCED* genes and simultaneously promoted the accumulation of sugars and anthocyanins. This pattern is consistent with previous evidence that sucrose stimulates ABA biosynthesis through induction of FaNCED1 and thereby contributes to the activation of the ripening program [15,16]. In addition, Jia et al. identified ABA-stress-ripening (ASR) as a potential convergence point of ABA and sucrose signaling [14]. *FaASR* is induced by both ABA and sucrose and can regulate downstream sugar transporter genes, providing a mechanistic framework for the coordinated regulation of carbohydrate accumulation and ripening.

The induction of FaSUT genes observed here is particularly important because sucrose accumulation depends not only on sucrose synthesis but also on its transport and compartmentalization. Recent studies have shown that *FaSnRK1α* positively regulates sucrose accumulation by affecting sucrose metabolic genes and sucrose transporter genes, including *FaSUT1* and *FaSUT5* [41]. More recently, *FaSWEET9a* was identified as a sucrose transporter regulated by the transcription factor *FaDOF2*, further highlighting the importance of transcriptional control of sugar transport in strawberry fruit [42]. Thus, the enhanced expression of *FaSUT* genes following ABA and sucrose treatments in the present study may represent an important mechanism for strengthening source-to-sink carbon allocation during fruit maturation. Interestingly, sucrose treatment induced a stronger increase in sugar accumulation than ABA treatment, suggesting that sucrose may influence ripening through both ABA-dependent and ABA-independent pathways. This interpretation is consistent with previous evidence showing that sucrose can activate ASR expression even when ABA biosynthesis is inhibited [14]. Therefore, sucrose may function as an integrative metabolic signal that simultaneously provides carbon skeletons for anthocyanin and organic acid metabolism and activates transcriptional programs associated with ripening.

Fruit softening is another major physiological hallmark of strawberry ripening. Strawberry softening results from coordinated modification of pectin, cellulose, hemicellulose and associated structural proteins rather than from the activity of a single enzyme [43]. Previous studies have shown that the degradation of covalently bound pectin and modifications of the pectic network are closely associated with strawberry softening [44,45]. The present results are therefore consistent with a model in which ABA and sucrose accelerate ripening-associated cell-wall remodeling, resulting in increased cell separation and tissue porosity. PG appears to be particularly important in this process. Although PG was historically considered to have a relatively minor role in strawberry softening because of its comparatively low activity in ripe fruit, genetic suppression of FaPG1 resulted in firmer fruits and increased retention of covalently bound pectins [46]. More recent studies have further demonstrated that PG-mediated pectin modification contributes substantially to strawberry fruit softening [47]. The increasing PG activity observed during fruit development in the present study, together with its further enhancement by ABA and sucrose, therefore provides physiological evidence that accelerated hormonal ripening is associated with increased pectin depolymerization.

PME modifies the methylesterification status of homogalacturonan, thereby altering the susceptibility of pectin to subsequent degradation. Draye and Van Cutsem showed that strawberry ripening involves marked changes in acidic pectin and PME-associated cell-wall remodeling [44]. Therefore, the increased PME activity observed under ABA and sucrose treatment may facilitate subsequent pectinolytic degradation, even though PME activity itself does not necessarily increase monotonically during ripening. The cellulase response is also consistent with the established role of auxin in maintaining cell-wall integrity. Harpster et al. identified an auxin-repressed endo-1,4-β-glucanase gene in strawberry whose expression increased at the onset of ripening and was suppressed by exogenous auxin [48]. Similarly, expansin genes such as *FaEXP1*, *FaEXP2* and *FaEXP5* have been associated with strawberry softening [49,50]. Consequently, the ability of IAA to maintain tissue compactness and suppress cell-wall degrading activities in the present study may contribute directly to its ripening-inhibitory effect. Recent evidence further supports the concept that hormonal signals regulate cell-wall remodeling through transcriptional networks. FaRIF, an NAC transcription factor, has been shown to coordinate ABA biosynthesis and signaling with cell-wall degradation, sugar accumulation and anthocyanin biosynthesis [51]. More recently, FaERF6 was reported to activate FaPG1 and promote strawberry fruit softening [52].

The expression profiles of *FaNCED1*, *FaNCED2*, *FaNCED3*, *FaABI1*, *FaSnRK2.6*, *FaMADS1* and other ripening-related genes provide molecular evidence supporting this interpretation. Most of these genes showed low expression during SG–LG but were strongly induced during WT–FR, and their expression was further enhanced by ABA and sucrose. This developmental pattern closely resembles the ABA-dependent ripening program described in previous transcriptomic studies, in which ABA controls a large proportion of genes activated during strawberry maturation [53,54]. The induction of FaSnRK2.6 is particularly consistent with the canonical ABA signaling pathway. ABA perception activates the PYR/PYL–PP2C–SnRK2 signaling module, ultimately leading to activation of ABA-responsive transcription factors and downstream ripening-related genes [55]. The induction of FaMADS1 is also of interest because MADS-box transcription factors have been implicated in the regulation of strawberry ripening. Previous studies have demonstrated that FaMADS1 and FaMADS2 respond to ABA and exhibit stage-dependent expression patterns during fruit development [56]. Similarly, the NAC transcription factor FaRIF regulates multiple aspects of strawberry ripening, including ABA metabolism, anthocyanin accumulation, sugar metabolism and cell-wall modification [51]. These findings suggest that the ripening response observed in the present study may arise from the coordinated activation of several transcriptional regulators rather than a single linear ABA pathway.

Furthermore, while strawberry is traditionally classified as a classic non-climacteric fruit, accumulating evidence indicates that it retains physiological sensitivity to ethylene during specific ripening phases [57,58]. Ethylene signaling components, such as FaETR receptors and FaEIN3/EIL transcription factors, exhibit distinct temporal expression patterns across receptacle development [59]. Rather than acting as the sole primary driver, ethylene signaling integrates into a complex cross-talk network with ABA and sucrose pathways. Recent studies demonstrate that ethylene can trigger ABA accumulation by modulating key microRNAs or inducing *FaNCED* expression, thereby fine-tuning late-stage physiological events such as cell wall degradation, color transition, and aroma volatile emissions [57]. More importantly, strawberry ripening is strongly associated with ABA, auxin and sugar signaling [53,54,60]. Importantly, the present study extends this model by demonstrating that these signals are closely associated with distinct cellular and metabolic processes. IAA is associated predominantly with the growth phase, whereas ABA and sucrose jointly promote the maturation phase characterized by anthocyanin accumulation, sugar enrichment and tissue softening. Overall, our findings suggest that the IAA–ABA–sucrose relationship represents a regulatory axis connecting cell expansion, hormonal transition, anthocyanin biosynthesis, sugar accumulation and cell-wall remodeling. The central feature of this network is the developmental decline of IAA and concomitant increase in ABA and sucrose. Rather than acting independently, ABA and sucrose appear to reinforce each other to establish the transcriptional and metabolic state required for ripening, whereas IAA antagonizes this transition by maintaining the early developmental program. This framework provides a useful basis for further dissecting the molecular links among hormone perception, sugar signaling and transcriptional regulation in non-climacteric fruit ripening.

## 5. Conclusions

In summary, this study systematically demonstrates the stage-specific coordination of IAA, ABA, and sucrose signaling in regulating strawberry fruit development and ripening. High endogenous IAA levels during early stages (SG–LG) promote cell expansion while maintaining compact tissue structure, limiting ABA accumulation, anthocyanin biosynthetic gene expression, sugar accumulation, and cell-wall degrading enzyme activities, thereby delaying the onset of ripening. As development progresses to the white and red stages (WT–FR), the decline in IAA coincides with a sharp rise in ABA and sucrose. Under these later stages, exogenous ABA and sucrose were associated with intercellular space enlargement and tissue softening (via elevated PG, PME, and cellulase activities), anthocyanin biosynthesis (through coordinated upregulation of *FaCHS*, *FaCHI*, *FaF3H*, *FaDFR*, *FaANS*, *FaUFGT*, etc.), soluble sugar accumulation, and the expression of key ripening-related genes (*FaNCEDs*, *FaSnRK2.6*, *FaSUTs*, etc.), whereas exogenous IAA produces largely opposite effects. These observations are consistent with a developmental model in which the decline of IAA and the concomitant rise of ABA and sucrose are associated with the transition from growth to ripening programs in non-climacteric strawberry fruit. Formal tests of antagonism, synergy, or treatment × developmental-stage interactions were not performed; therefore, the present conclusions are limited to the observed opposing and parallel response patterns. Collectively, the findings provide a theoretical foundation for targeted hormonal and metabolic interventions aimed at optimizing fruit quality.

## Figures and Tables

**Table 1:** Effects of ABA, IAA and sucrose on the color index difference of strawberry fruits.

	Anthocyanin Content (mg/Kg)	L	a*	b*	C	H	CIRG
Untreated Control	53.11 ± 4.69	44.46 ± 3.98	34.89 ± 4.96	21.73 ± 1.99	41.10 ± 3.96	31.92 ± 2.96	1.73 ± 0.17
Water	78.72 ± 6.68 *	40.35 ± 3.69	39.91 ± 4.65 *	20.17 ± 2.46	44.71 ± 5.02 *	26.81 ± 2.76 *	1.80 ± 0.16
50 μM ABA	161.72 ± 9.36 **	32.71 ± 3.66 **	33.49 ± 3.56	13.9 ± 1.66 **	36.26 ± 4.11 *	22.54 ± 2.36 *	2.28 ± 0.24 *
30 mM Sucrose	119.98 ± 10.25 **	34.09 ± 2.98 *	29.41 ± 3.05 *	12.65 ± 1.45 **	32.02 ± 3.19 *	23.27 ± 2.45 *	2.37 ± 0.24 *
20 μM IAA	20.75 ± 1.99 *	56.65 ± 4.98 **	20.28 ± 2.22 **	23.56 ± 2.45	31.09 ± 2.96 *	49.28 ± 5.03 **	1.49 ± 0.16 *

Values are means ± SD of four biological samples (each sample included ten fruits). Asterisks indicate two-sided unpaired Student’s *t*-test versus the untreated control: * *p* ≤ 0.05; ** *p* ≤ 0.01.

## Data Availability

The datasets supporting the results of this article are included within the article and additional Supplemental Materials.

## Supplementary Materials

The following supporting information can be downloaded at: https://www.mdpi.com/article/10.3390/plants15213314/s1, Table S1: Primer pairs of RT-qPCR.
